# Small intestinal diverticulum with bleeding: Case report and literature review

**DOI:** 10.1097/MD.0000000000009871

**Published:** 2018-03-02

**Authors:** Lifang Zhao, Wei Lu, Yinping Sun, Junrong Liang, Shanshan Feng, Yongquan Shi, Qiong Wu, Jianhong Wang, Kaichun Wu

**Affiliations:** a Emergency Room of Digestive Diseases, National Clinical Research Center for Digestive Diseases and Xijing Hospital of Digestive Diseases, Fourth Military Medical University, Xi’an; b The Outpatient Internal Medicine Department of Beijing Veteran Cadre Service Administration, Central Military Commission Logistics Support Department, Beijing, China.

**Keywords:** computed tomographic enterography, obscure gastrointestinal bleeding, small intestine diverticulum, small intestine endoscope

## Abstract

**Rationale:**

Small intestinal diverticulum with bleeding is an important reason for obscure gastrointestinal bleeding (OGB) , in addition to tumor and vascular diseases. Small intestinal diverticulum with bleeding is difficult to detect by barium meal and angiographic methods and has been regarded as an important cause of obscure gastrointestinal tract bleeding in adolescents. Because of its complicated etiology and non-specific clinical manifestations, it is relatively difficult to detect small intestinal diverticulum with bleeding, especially in patients with a large amount of bleeding and hemodynamic instability.

**Patient concerns:**

This retrospective study collects clinical statistics of 19 patients admitted to our hospital from January 2010 to December 2016. Patients who had small intestinal diverticulum patients with bleeding were included in this study. Patients who were taking anticoagulants were excluded

**Diagnoses:**

Small intestinal diverticulum patients with bleeding.

**Interventions:**

This retrospective study describes the clinical features of patients with small intestinal diverticulum whose main symptom was gastrointestinal bleeding and analyze the literature on this topic, with particular reference to the clinical characteristics, pathological features, and choice of examination methods.

**Lessons:**

Small intestinal diverticulum with bleeding is a common cause of obscure gastrointestinal bleeding, but it is difficult to detect using normal examination methods. For patients with repeated gastrointestinal bleeding and no positive results found on gastroscopy and colonoscopy, endoscopy of the small intestine and CTE with contrast can be considered as a diagnostic modality.

## 1 Introduction

Obscure gastrointestinal tract bleeding is a rare clinical disease, accounting for 5% of gastrointestinal tract bleeding,^[1]^ which is often caused by small intestine bleeding. Small intestinal diverticulum with bleeding is an important reason for obscure gastrointestinal bleeding (OGB), in addition to tumor and vascular diseases.^[2]^ Small intestinal diverticulum with bleeding is difficult to detect by barium meal and angiographic methods and has been regarded as an important cause of obscure gastrointestinal tract bleeding in adolescents.^[3]^ Because of its complicated etiology and nonspecific clinical manifestations, it is relatively difficult to detect small intestinal diverticulum with bleeding, especially in patients with a large amount of bleeding and hemodynamic instability.

We have experienced small intestinal diverticulum patients with bleeding in our emergency room; thus, herein, we further describe the clinical characteristics of small intestinal diverticulum with bleeding in these patients and discuss the features and proper examination methods for small intestinal diverticulum with bleeding.

## 2 Patients and methods

Small intestinal diverticulum is an important cause of small intestine bleeding, and as the clinical symptoms are not always typical, this condition cannot be easily detected by normal gastroscopy and colonoscopy. This retrospective study collects clinical statistics of 19 patients admitted to our hospital from January 2010 to December 2016. Patients who had small intestinal diverticulum patients with bleeding were included in this study. Patients who were taking anticoagulants were excluded. This study was performed according to the Declaration of Helsinki, and approved by the institutional review board at Xijing Hospital. The informed consents were obtained from the patients.

## 3 Results

### 3.1 Patient characteristics

Among the 19 patients, 18 were men and 1 was a woman. The age range was 15 to 60 years, with an average age of 27 years. Fourteen patients were in the age range between 16 and 30 years. The initial symptoms were different among the patients: 15 patients had blood in their stool, 2 had black stool, and another 2 had black stool with hematemesis. Among them, 9 patients had associated symptoms: 5 patients had abdominal pain, 2 patients had bloating, 1 patient had syncope, and 1 patient had shock. Fourteen patients had repeated gastrointestinal bleeding, and another 5 patients had initial bleeding. Four patients had a history of blood transfusion, 2 patients had undergone appendectomy, and 1 patient had high blood pressure.

The onset of symptoms was 10 hours to 11 years. When patients came to the clinic, the blood pressure range was 90 to 132/60 to 80 mm Hg and heart rate range was 60 to 98 beats/min with an average rate of 77 beats/min. The auxiliary examination showed the following: red blood cell count 1.6 to 5.16 × 10^9^/L (average 3.29 × 10^9^/L), hemoglobin level 54 to 137.6 g/L (average 87.5 g/L), platelet count 95 to 264 × 10^9^/L (average 166.4 × 10^9^/L), total protein level 37.0 to 70.6 g/L (average 55.9 g/L), and albumin level 25 to 47.6 g/L (average 36.8 g/L). Among the 19 patients, 9 had positive occult blood in their stool, and another 10 patients had negative occult blood.

### 3.2 Examination results

**Figure 1:**
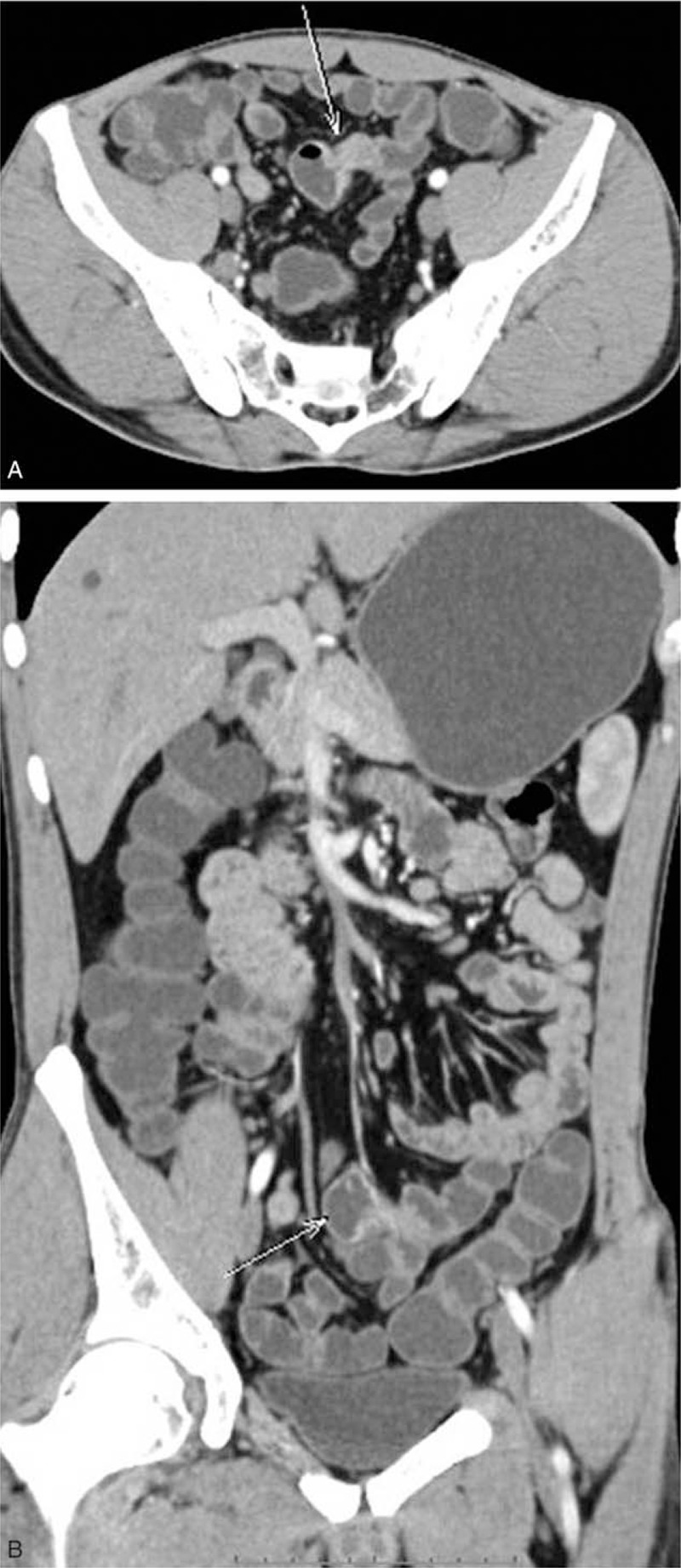
Small intestinal diverticulum is examined by CTE with contrast. (A and B) The arrow indicates the pouch-like protrusion, and a blind end is formed in the pelvic ileum. The intestinal wall is slightly enhanced, and liquid and gas shadows can be seen within the intestinal wall, suggesting the formation of local diverticulum in the small intestine.

All patients underwent 1 or 2 examination strategies, such as gastroscopy, colonoscopy, computed tomography (CT) enterography with contrast, endoscopy of the small intestine, wireless capsule endoscopy, or digital subtraction angiography. As summarized in Table 1, among the 15 patients who underwent gastroscopy, 1 patient was diagnosed as having duodenal diverticulum. Among the 12 patients who underwent colonoscopy, 2 patients were diagnosed as having bleeding above the ileocecum, and 1 patient was diagnosed as having ileocecal lump. Among the 10 patients who underwent a CT examination, 6 patients were diagnosed as having ileal diverticulum. The indicative CT result is shown in Fig. 1 A and B. The 2 patients who underwent emergency digital subtraction angiography had no abnormality. Among the 8 patients who underwent endoscopy of the small intestine, 5 were diagnosed as having diverticulum. In addition, the 2 patients who underwent wireless capsule endoscopy had no abnormality.

**Table 1:**
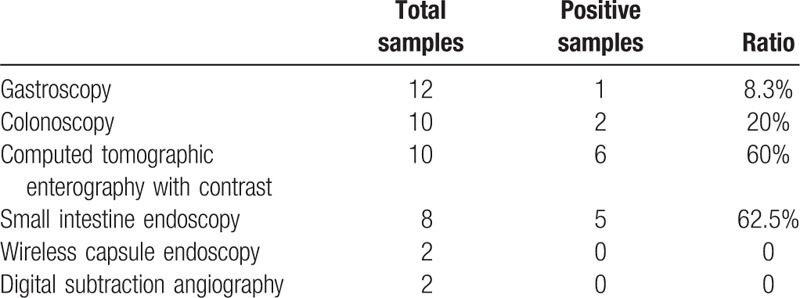
Comparison of different examining methods for positive ratio in patients with small intestine diverticulum with bleeding.

### 3.3 Surgical results

All 19 patients with small intestinal diverticulum underwent surgical management; the operative time was 60 to 230 minutes with an average of 115 minutes. Two patients received a blood transfusion. Among the 19 patients, 2 had duodenal diverticulum, 2 had jejunal diverticulum, 3 had ileal diverticulum, and 12 had Meckel diverticulum. The smallest diverticulum removed measured 3 × 1 × 2 cm, and the largest one measured 15 × 5 × 3 cm.

### 3.4 Pathological results

**Figure 2:**
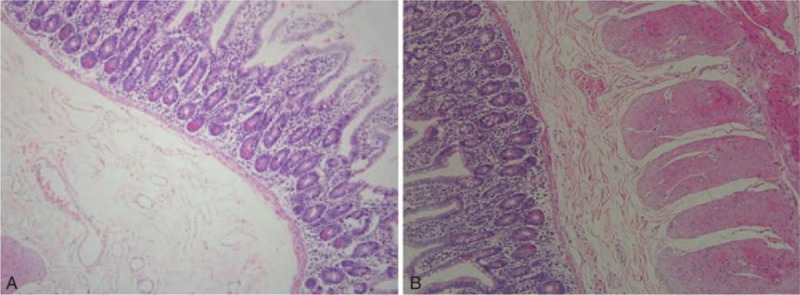
Indicative pathological results for ectopic gastric mucosa and ectopic pancreas in small intestinal diverticulum. (A) Ectopic gastric mucosa is found in a patient with small intestinal diverticulum. (B) Ectopic pancreas is found in a patient with small intestinal diverticulum.

All surgical specimens were confirmed by pathological examination as diverticulum. Among them, 8 patients were diagnosed as having diverticulum with ectopic gastric mucosa (the indicative picture is shown in Fig. 2A); 2 patients were diagnosed as having diverticulum with ectopic pancreas (the indicative picture is shown in Fig. 2B); 2 patients were diagnosed as having diverticulum with ectopic gastric mucosa and ectopic pancreas; and 6 patients were diagnosed as having diverticulum with chronic mucous membrane inflammation.

### 3.5 Follow-up

All patients were followed up for 3 months to 4 years by telephone or outpatient follow-up. One of the 19 patients developed pancreatic leakage, underwent medical treatment, and was healing without bleeding. Another patient had blood in the stool 1-half year postoperatively, but no abnormality was detected by endoscopy and dual-source CT of the small intestine. He was healing after medical treatment.

## 4 Discussion

Small intestinal diverticulum includes duodenal diverticulum, Meckel diverticulum (diverticulum of the distal ileum), and jejunal diverticulum, and this condition can be divided into 2 subgroups: congenital and acquired small intestinal diverticulum. Usually, small intestinal diverticulum does not manifest with any symptoms, except for complications, such as inflammation, bowel obstruction, and gastrointestinal bleeding.^[4]^ Bleeding in small intestinal diverticulum can be black or bloody stool can be present for a long time without obvious causes. In the present study, 14 of 19 patients had repeated gastrointestinal bleeding, with the longest duration of 11 years. Shock can occur when bleeding is profuse, and in this study, 2 patients developed shock. Previous studies showed that Meckel diverticulum was the most common type in small intestinal diverticulum^[5]^; in addition, symptomatic diverticulum was more common in male patients than in female patients and more common in younger patients than in older ones.^[6]^ The present study also suggested that small intestinal diverticulum patients with bleeding are usually men and young people, which is consistent with the finding of a previous study.^[7]^ Therefore, for young, male patients with repeated unexplained bleeding, small intestinal diverticulum may be considered the cause of bleeding.

Meckel diverticulum is a common congenital intestinal malformation caused by embryonic dysplasia.^[8]^ Small intestinal diverticulum was first described by Hidaus in 1958, but Meckel diverticulum was named by Johann Friedrich Meckel the Younger in 1809^[9]^ and identified to be true diverticulum because it usually involves the whole layer of the small bowel.^[10]^ It was found that in the early stages of embryonic development, the yolk tube is located between the midgut and the yolk sac; subsequently, the yolk tube gradually shrinks into the fiber cord, and then completely degrades without any traces. If the degradation is not completed, different types of deformity can remain^[11,12]^; the most common one is descent of the yolk tube linked with the umbilical, while the small bowel of the yolk tube is still connected to the intestine, which forms Meckel diverticulum.^[13]^ It was reported that Meckel diverticulum usually occurs within 100 cm from the ileocecal valve.^[14]^ However, in the present study, Meckel diverticulum in 12 cases was located at sites 50 to 150 cm from the ileocecal valve.

Meckel diverticulum is the residual body of the yolk tube, and cells in the yolk tube have the potential to differentiate into various types of mucosa; thus, it is easy to find ectopic tissues in the diverticulum, with the most common types being gastric mucosa, pancreatic cells, and intestinal mucosa.^[15,16]^ The ectopic mucosa also has the ability to secrete digestive enzymes, such as gastric acid and other chemical substances, which cause the formation of ulcers of adjacent mucosa and even bleeding.^[17–19]^ In the present study, 8 of 19 patients with diverticulum had ectopic gastric mucosa, 2 had ectopic pancreatic mucosa, and 2 had both; all the patients had symptoms of bleeding.

Complications such as small intestinal obstructions and intussusceptions can occur in the cases that some small intestine flaps form around Meckel diverticulum or fibers exist between the small intestine and the umbilicus. Meckel diverticulum is usually asymptomatic with a 4% to 40% risk of complication during the patient's lifetime.^[20,21]^ Actually, the main clinical symptoms of the diverticulum usually manifest in the form of complications: bleeding, intestinal obstruction, diverticulitis, perforation, stone formation, and neoplasm.^[21]^ According to a survey by the Mayo clinic,^[22]^ patients with diverticulum are prone to having complications when the length of the diverticulum is greater than 2 cm. In the present study, all 19 patients had bleeding, and all patients’ diverticulum was greater than 2 cm long.

The diagnosis of diverticulum in the duodenal part mainly depends on gastroscopy or pancreatic duct cholangiography due to the specific anatomical position of the duodenum. However, the small intestine is long with curvatures and overlaps; thus, it is difficult to examine Meckel diverticulum and the diverticulum of the ileum. Gastroscopy, colonoscopy, and ultrasonography lack specificity to the small intestine and can be only used to exclude gastrointestinal bleeding from other sites. The whole digestive tract barium meal cannot provide the entire view of the small intestine or obtain integrative imaging of the small intestine; as it sometimes can even induce intestinal obstruction, it can also be excluded from examination of diverticulum. Although it was reported that wireless capsule endoscopy is a useful diagnostic tool for diagnosing Meckel diverticulum,^[23,24]^ the camera can sometimes become stuck in the intestine^[25,26]^ and cause dangerous conditions^[27]^; thus, wireless capsule endoscopy might not be the first choice for examining small intestinal diverticulum.^[28]^ Compared with capsule endoscopy, double-balloon enteroscopy is reported to be a safer and more effective method; in addition, as it can be used to examine small intestinal diverticulum from both oral and anal directions and the small bowel completely, it can determine the specific location of the small intestinal lesions.^[29–31]^

Studies have shown that double balloon enteroscopy (DBE) can not only accurately detect small intestinal diverticulum with a typical diverticulum structure, but it can also determine whether bleeding is caused by diverticulum and where the diverticulum is located; therefore, results from the DBE examination can guide the surgical treatment.^[28,32]^ However, when the amount of bleeding is large, it is rather difficult to see the lesions using DBE, and it is difficult to perform DBE when the patient's hemodynamic status is not stable. Furthermore, it was reported that DBE is an invasive examination strategy, and it sometimes can cause serious complications, such as perforation, bleeding, and acute pancreatitis.^[33]^

CTE with contrast is another strategy for examining small intestinal diverticulum.^[34]^ CTE with contrast usually shows a pouch protruding outside the small intestine wall at the margin of the small mesenteric vein. The pouch usually oppresses the surrounding tissues with liquid or a sieve-like low-density shadow. In the cases with bleeding, the contrast agent can be seen inside the diverticulum in the arterial phase and disperse into the surroundings in the venous phase. CTE can not only show the shape of the diverticulum but can also show the diverticulum cavity and adjacent abnormal structural changes.^[8,35]^ However, the detection is related to the amount of peritoneal fat; thus, CTE should not be used in patients with a high body mass index.^[36]^

## 5 Conclusion

Small bowel diverticulum patients with bleeding usually lack specific clinical symptoms and typical signs. DBE can intuitively detect lesions of small intestinal diverticulum, and its results can guide subsequent surgical treatment. However, for patients with a large amount of bleeding and hemodynamic instability, DBE can increase the risk and affect the observation of lesions; therefore, the present study suggests that in small bowel diverticulum patients with bleeding, CTE with contrast should be used as a promising method to help diagnosis. Despite the positive discovery of the study, some limitations should not be ignored. Some studies use MRE for the diagnosis for small bowel diverticulum patients with bleeding; however, the present study did not compare the usefulness of MRE with CTE. Future studies should compare the positive detection rate and the usefulness of MRE and CTE for the future instructions of small bowel diverticulum patients with bleeding.
